# Participatory development of a manual for the implementation of diversity-sensitive palliative and hospice care in Germany: a mixed-methods study protocol

**DOI:** 10.1186/s12904-023-01252-y

**Published:** 2023-09-05

**Authors:** Fabian Erdsiek, Yüce Yılmaz-Aslan, Patrick Brzoska

**Affiliations:** Health Services Research, Faculty of Health, School of Medicine, Witten/Herdecke University, Alfred-Herrhausen-Straße 50, 58448 Witten, Germany

## Abstract

**Background:**

The diversity of the population is associated with different needs and expectations towards palliative and hospice care. Current approaches available in Germany generally fall short in addressing the role of diversity and intersectionality in this health care setting and healthcare facilities struggle with organizational difficulties and missing information on how to implement corresponding diversity-sensitive measures. The present study aims to develop a hands-on manual that enables providers of hospice and palliative care to implement measures and strategies for diversity-sensitive care, while taking into account the perspective of healthcare users and explicitly including vulnerable and minority patient groups.

**Methods:**

A participatory approach is used to co-create the aforementioned manual using an explanatory sequential mixed-methods design. First, based on a systematic analysis of existing measures, an initial draft of the manual will be developed. Subsequently, an online survey will be conducted among all hospice and palliative care providers in Germany (n = 2,823). Based on the results of the survey, 12 to 15 qualitative problem-centered interviews will be conducted with employees of selected providers who took part in the survey. Results of the survey and the qualitative interviews will be integrated and analyzed. In parallel to the development and research process, a comprehensive dissemination strategy will be developed.

**Discussion:**

The manual will assist providers of palliative and hospice care in determining goals, needs, and available resources in order to utilize patient-centered and diversity-sensitive measures to meet a wide range of expectations. It can also be informative for providers in other countries. The participatory co-development approach ensures the practical relevance of the manual, while the mixed-methods design allows for targeted input on the manual’s usability, acceptance, and viability as a supportive tool.

## Background

Increased international migration movements and globalization tendencies have led to a diversification of lifestyles and personal values. Diversity dimensions such as age, gender identity, income, educational attainment, or physical and psychological disabilities are associated with different needs and expectations regarding the utilization of health services. In a similar vein, health service use varies between people of different nationalities or origin. In Germany, currently, about 21.9 million residents have a migration background, defined as having migrated to Germany themselves or having at least one parent who immigrated to Germany [1]. Migrants in Germany differ from the majority population in several health aspects, e.g. with regard to a higher prevalence of several non-communicable diseases, such as cardiovascular diseases, type 2 diabetes mellitus, chronic pain, and certain psychological conditions [2–5]. The majority of studies on early cancer detection in Germany have further shown that migrants are less likely to use screening services for early detection of skin, cervical, and colon cancer [6–8], while participation rates for mammography screening were generally higher among women with a Turkish migration background and German resettlers from Eastern Europe [9, 10]. Similarly, migrants are less likely to use hospice or palliative care services [11, 12]. In addition to more traditional concepts of family and care that may reduce the need or interest in such services [13–15], barriers that prevent or impede the use of care can be found in the hospice and palliative care sector as well [16, 17]. Information deficits, language barriers, the complexity of the care sector, as well as insufficiently addressed cultural differences relating to illness and dying can negatively influence service utilization [14, 17, 18].

In addition, interactions between different diversity dimensions (intersectionality) can influence the encounters or effects of barriers to health service use [19]. One study found that in Berlin, people with a Turkish background were underrepresented in local facilities for hospice and palliative care, while people with a Polish or Russian background were overrepresented compared to their respective shares in the Berlin population [20]. This shows that strategies addressing the needs of people with a migration background indiscriminately and without consideration of other dimensions of diversity are insufficient.

Current approaches available in Germany generally fall short in addressing intersectionality and diversity and focus solely on migration status or are limited to specific problem situations, e.g., neutral prayer rooms or multilingual information materials [11]. Other, more recent approaches use narrow concepts of culture and cultural competence to provide a more differentiated perspective on care, while generally disregarding gender aspects, physical or psychological disabilities, and other diversity dimensions [21, 22]. While a more recent guide offers some advice to better address the needs of vulnerable populations, this guide does not provide any support to implementing related strategies [23]. Studies have shown that aside from financial considerations, many healthcare facilities struggle with organizational difficulties and missing information on how to implement corresponding diversity-sensitive measures [24, 25]. Therefore, the *ParDiMi* project, funded by the German Cancer Aid (Deutsche Krebshilfe), aims to develop a hands-on manual that enables providers of hospice and palliative care to implement measures and strategies for diversity-sensitive care, while taking into account the perspective of healthcare users and explicitly including vulnerable or minority patient groups.

## Methods

**Fig. 1:**
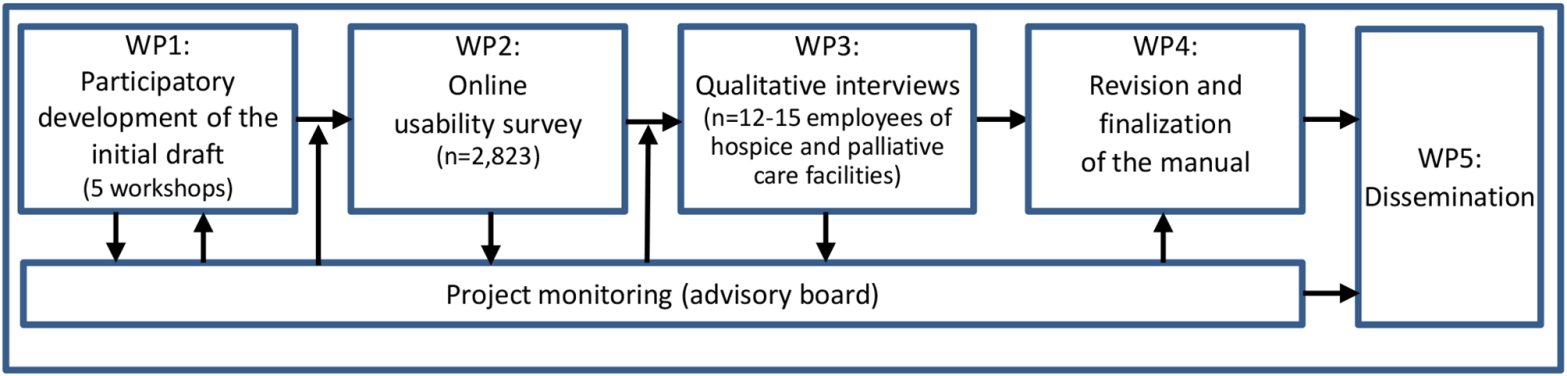
Project structure and work packages (WP)

*ParDiMi* uses a participatory approach to co-create a manual for healthcare professionals in the palliative and hospice care sector seeking to make their facilities and services responsive to diversity. This will be conducted over the course of 30 months using an explanatory sequential mixed-methods approach (Fig. 1). Based on prior research and a systematic analysis of existing measures and strategies for diversity-responsive or diversity-sensitive care, first, an initial draft of the manual will be developed. This draft version will then be made available to all German hospice and palliative care providers listed in the German “Guide to hospices and palliative care” [26]. An online survey among these service providers will be conducted to assess acceptability and usability as well as to identify potential shortcomings and possible improvements. Based on the results of the survey, qualitative interviews with representatives of care providers will be carried out to gain a deeper understanding of the identified shortcomings and with regard to how to revise the manual. Based on the results of the survey and the qualitative interviews, a revised version of the manual will be developed and finalized. In parallel to these steps, a dedicated communication and dissemination strategy will be developed and carried out to increase the visibility of the project, heighten awareness and promote participation in the study.

### Participation

**Fig. 2:**
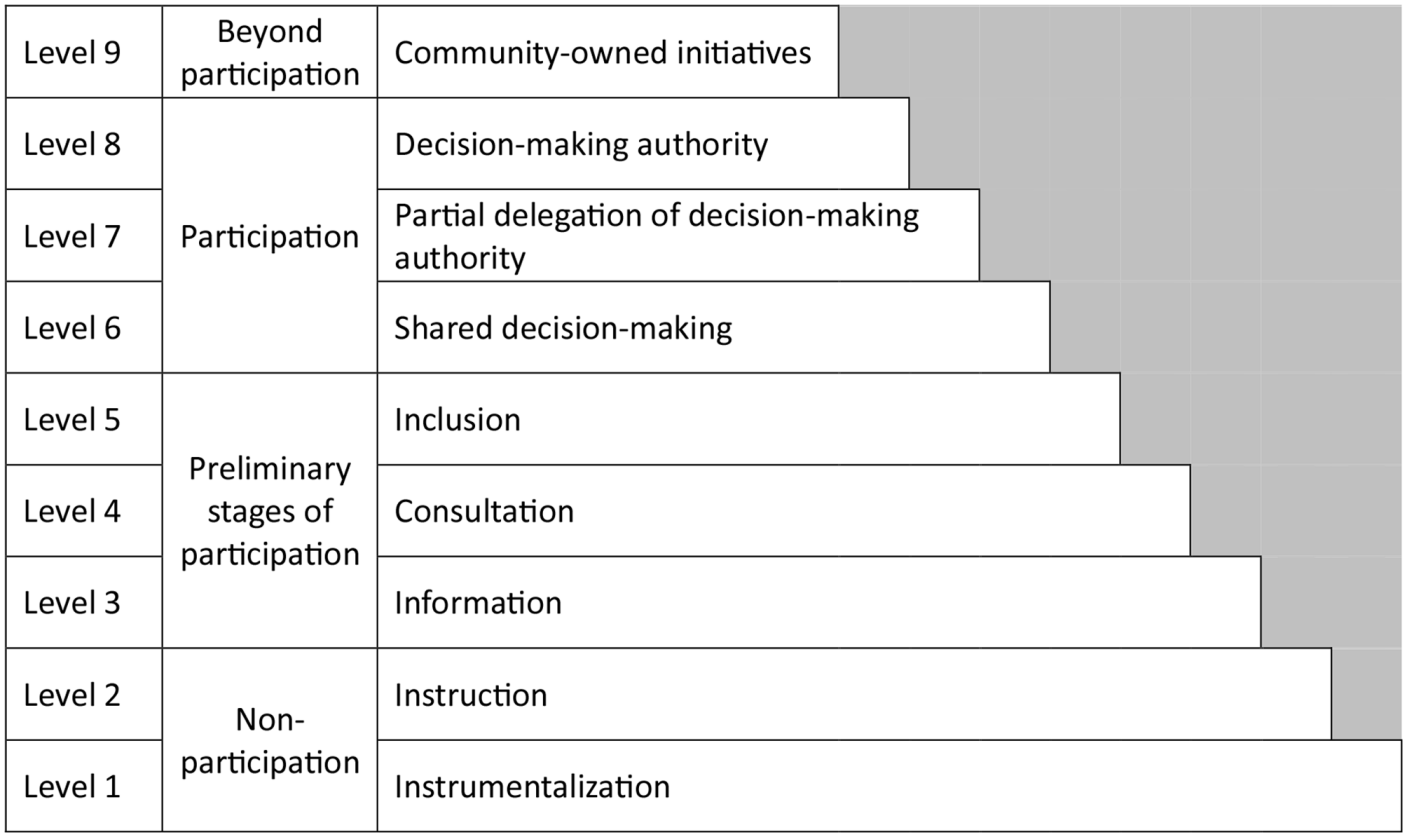
Levels of participation (own illustration based on Wright et al. 2010)

In the scope of this project, participation of different stakeholders in the development of the manual is a major component. To identify important stakeholders, to determine the overall level of participation and to get an initial understanding of main areas of interest for different key stake holders, a focus group discussion with experts and patients was carried out during the initial planning stages [27]. Based on the results of the focus group discussion, main stakeholders to be involved were determined to be patients, relatives of patients, representatives of patient organizations (self-help groups, etc.), palliative and hospice care experts and care providers (specifically doctors, nurses and other directly care-related professions). The overall level of participation intended was determined to be “Shared Decision-Making” (Fig. 2) as defined in the Levels of Participation model [28].

For the *ParDiMi* project, patients, relatives of patients, and employees of care providers will be involved as co-researchers and co-creators where reasonably possible in all steps of the project. Palliative and hospice care experts, representatives of patient organizations and management-level representatives of palliative and hospice care providers, in addition, will be part of a dedicated advisory board that will provide feedback, coordinate with researchers and co-researchers for the development and execution of the dissemination strategy and provide additional quality control.

All co-researchers and members of the advisory board will be involved according to their own preferred level of involvement. In addition, multiple ways of involvement will be offered, including online and telephone conferences, online access to the draft versions of the manual and all relevant project documents (questionnaires, interview guides, press releases, etc.). Co-researchers and advisors will be free to reduce or increase their level of participation at any point in the process according to their own preference. These measures serve to enable self-determined participation and address potential limitations in participation due to health concerns or other factors that could impact the ability or motivation to participate.

### Mixed methods-design

For the study we chose an explanatory sequential mixed methods study design [29]. Based on prior research and the feedback of the advisory board, an initial draft of the manual will be developed. This feedback will also inform the structure of the questionnaire for the usability survey. The results from the online survey will be used to refine the sampling strategy and the development of the interview guide for the qualitative interviews. In contrast to triangulation designs, where a single research subject is analysed from a qualitative and quantitative perspective, explanatory sequential designs present the opportunity to broaden the scope of the research based on the initial findings and therefore to gather additional information on specific aspects [30]. The results of the quantitative and qualitative study parts will guide the revision and finalization of the manual. Over the course of the project, a dedicated dissemination strategy will be developed and carried out to increase participation in the survey and interviews, raise awareness for the project, and promote the final manual.

### Work packages

#### WP1

The research team and the co-researchers will jointly develop an initial draft of the manual using an iterative process over the course of 10 months. To facilitate joint development, 5 bi-monthly workshops will be conducted. In addition, co-researchers will receive regular updates on the project progress as well as online access to all necessary documents and files for the initial draft to enable them to contribute and participate. The first workshop will serve to define key topics and central elements that are essential to diversity-sensitive care and need to be included in the manual. Additionally, the workshop will be used to introduce the co-researchers into basic aspects of scientific research and public health. Subsequently, a preliminary outline for the structure of the manual, together with a rough outline of the contents for each chapter, will be developed. The second workshop will be used to discuss and consent the outline for the structure and the main contents of the different sections, as well as to train the co-researchers in basic principles of quantitative health research. Subsequently, the different sections of the manual will be jointly written by the researchers and co-researchers. In addition, two more workshops will be conducted to discuss and finalize the contents and structure of the different sections of the manual. These workshops are also meant to provide a way to contribute for co-researchers who feel overwhelmed by or unable to participate in the entire development process of the chapters. Next, the initial draft will be put together, additional information will be added, and a preliminary layout will be developed. The last workshop of WP1 will be used to reach consensus on the final layout and structure of the manual before the quantitative online survey. The project advisory board will be involved in the first and fourth workshop, in order to provide an outside expertise and a first input for the development of the survey questionnaire.

#### WP2

The draft version of the manual will be made available online. Subsequently, an online survey will be conducted among German hospice and palliative care providers. Providers will be asked to provide feedback on selected indicators of usability and implementation [31]. Researchers, co-researchers and the advisory board will jointly decide on which instruments or parts thereof will be included in the survey. Preliminary research has narrowed down potential instruments to the Intervention Usability Scale (IUS) [32], and the German versions of the Acceptability of Intervention Measure (AIM), the Intervention Appropriateness Measure (IAM), and the Feasibility of Intervention Measure (FIM) [33, 34]. The survey will be a representative nation-wide online survey of all providers (n = 2,823) listed in the German “Guide to hospices and palliative care” [26], including hospices (n = 249), palliative wards (n = 329), palliative care, mobile palliative teams in hospitals (n = 76), outpatient hospice care (n = 1,168), specialized outpatient palliative care teams (SAPV-teams) and palliative medical consultation services (n = 317), as well as outpatient palliative care services (n = 21), and resident palliative doctors (n = 663). To increase participation and avoid selection bias, providers will be contacted twice over the course of two months. Based on prior research, we estimate a response rate of 20%, leading to a final sample size of n = 565. Providers not interested in participating in the survey will be asked to take part in a short non-responder survey to identify differences between participants and non-participants and to better estimate representativity of the sample, including structural data of the provider (size, region, etc.) and demographic data of the respondents (e.g., age, gender, educational attainment). Descriptive analyses will be used to describe the sample and derive overall results. Additional multiple regression analyses will be conducted to identify potential confounders and associations between scores for usability, acceptability, and feasibility and structural aspects of the provider or demographic factors of the respondents. Interpretation of the results will be done jointly among researchers and co-researchers. The results and their interpretation will be presented to the advisory board and serve as the basis for the development of the interview guide used for the qualitative interviews.

#### WP3

Based on the results of the survey, qualitative problem-centered interviews will be conducted with employees of selected providers who took part in the survey. The aim of these interviews is to gain deeper information on open questions, on challenges using the manual, on barriers encountered in implementation and with respect to suggestions to revise the manual. In cooperation with the co-researchers, the researchers will develop an interview guide and provide it to the advisory board for feedback. For the recruitment of the interview participants, a purposive sampling approach with maximum variation will be used in order to capture a greater variety of perspectives [35]. Based on prior experience, we aim to conduct 12 to 15 interviews. The number of interviews is a preliminary estimation and could be increased or reduced depending on data saturation [36]. The interviews will be analyzed by the researchers using a thematic qualitative content analysis approach [37, 38]. For this purpose, an initial coding frame will be created based on the interview guide, supplemented by inductive coding of the data to derive further relevant codes and categories [38]. Each category will then be described through definitions and anchor examples. Categories, definitions, and anchor examples will be discussed by all researchers and co-researchers until consensus is reached.

#### WP4

Following WP3, results of the survey and the qualitative interviews will be integrated and analysed, using the results of the qualitative interviews to better interpret and contextualise findings from the survey [29, 30]. This analysis will allow a more comprehensive consideration of challenges and limitations of the usability and acceptability of the draft version and a concise revision process. Based on the results of the analysis, the researchers and co-researchers will revise the manual, using two further workshops. During the first workshop, necessary changes, additions, and modifications will be discussed. Following the workshop, the manual will be revised over a period of three months before, in the last workshop, the final version of the manual will be presented to all members of the project team and the advisory board.

#### WP5

In parallel to the development and research process, the researchers and co-researchers will develop and implement a comprehensive dissemination strategy. In cooperation with the advisory board, communication channels, relevant multipliers and further stakeholders will be identified. Since the survey (WP2) is designed to reach the vast majority of German providers of hospice and palliative care, an initial increase of awareness among the target audience can be reached and built upon. Over the course of the project, further information materials (e.g., flyers, brochures, handouts) will be developed and disseminated using suitable distribution channels (e.g., social media, newsletters) at different points in time to serve as reminders. Dissemination of the final version of the manual is a main goal of the project. The strategy for disseminating the manual will be devised over the course of the project using the participatory approach. In addition, central aspects or steps to introduce diversity-sensitive palliative and hospice care will be disseminated as a webinar using YouTube videos.

## Discussion

Addressing the varying needs and expectations of a diverse patient population is a central aspect of user-oriented healthcare. In order to address these needs and expectations in the hospice and palliative care setting, providers need to implement diversity-sensitive measures and strategies. Currently, providers interested in developing or implementing such measures and strategies are often lacking the knowledge to do so. *ParDiMi* is designed to develop a hands-on manual to support providers in developing and implementing diversity-sensitive strategies. The final manual will aid providers in identifying goals, needs, and available resources to develop feasible, user-oriented measures to address diverse needs and expectations. The participatory approach chosen for the development ensures user-orientation and practical relevance of the manual, while the mixed-methods design allows for dedicated feedback on the usability, acceptability, and feasibility of the manual and its contents as a supporting tool. Some limitations of the project need to be considered as well. In the scope of the project, a complete evaluation of the manual is not possible, suggesting the need for a follow-up study to assess the effectiveness of the manual and its potential long-term impact. In addition, the potential effects of the manual will at first likely be limited to hospice and palliative care facilities generally interested in providing diversity-sensitive care. Although the project includes a dedicated dissemination strategy that is meant to increase awareness for the importance of diversity-sensitive care, other studies have shown that acceptance of diversity aspects in the German health system is still limited [24, 25]. In addition, many healthcare facilities report a lack of incentives and insufficient financial resources as important barriers to implementing diversity-sensitive measures [24, 25]. These challenges demonstrate the need for further dedicated health policies that can also be informed by the results of this project.

## Data Availability

This study utilizes confidential patient data protected by the German Data Privacy Act. In order to ensure patient confidentiality and privacy, the data used in this study will only be available in anonymous and aggregated form and will be provided to researchers upon reasonable request.
